# Prediction of Fatigue Life of Polyetherimide/Carbon Fiber Particulate Composites at Various Maximum Stresses and Filler Contents

**DOI:** 10.3390/polym16060749

**Published:** 2024-03-08

**Authors:** Alexey A. Bogdanov, Sergey V. Panin

**Affiliations:** 1 Laboratory of Mechanics of Polymer Composite Materials, Institute of Strength Physics and Materials Science of Siberian Branch of Russian Academy of Sciences, 634055 Tomsk, Russia; ispmsbogdanov@gmail.com; 2 Department of Materials Science, Engineering School of Advanced Manufacturing Technologies, National Research Tomsk Polytechnic University, 634050 Tomsk, Russia

**Keywords:** fatigue, polyetherimide, hysteresis loop, carbon fiber, polymer composite, structure–property relationship

## Abstract

The objective of this research was to predict the fatigue behavior of polyetherimide-based composites loaded with short carbon fibers 200 μm long under cyclic loads. The weight fraction of the filler was 10, 20, and 30 wt.%, while the maximum stress in a cycle was 55, 65, and 75 MPa. A modified fatigue model based on the obtained experimental results and Basquin equation was developed. The novelty of the results is related to developing a model on the structure–property relationship, which accounts for both the maximum stress in a cycle and the carbon fiber content in the composites. In addition, an “algorithm” for designing such composites according to the fatigue life criterion was proposed. The approach to determine relationships between the composition, structure, and properties of PCMs described in this study can be applied to further expand the model and to improve its versatility in the use of other thermoplastic matrices and fillers. The results of this study can be applied for the design of composites for structural applications with designated fatigue properties.

## 1. Introduction

Currently, polymer composite materials (PCMs) are widely used in the aerospace, shipbuilding, and automotive industries, as well as in construction, medicine, etc. Sets of required physical and mechanical properties of the PCMs can include up to several dozen different parameters. Despite the fact that the number of commercially available PCMs is quite large, their characteristics gradually cease to meet the ever-tightening requirements as technologies are improved, prompting the development of new grades [1,2,3,4,5].

Typically, high-strength PCMs are developed through the reinforcement with continuous fibers. At the same time, dispersed filling is a more technologically advanced way for manufacturing products from PCMs based on thermoplastic binders, although it does not provide advanced mechanical properties. In addition, the need to improve various sets of functional characteristics of the PCMs operating under extreme conditions (including at elevated temperatures) determines the implementation of thermoplastic high-performance polymers (HPPs) instead of conventional thermoset matrices [6,7,8,9,10].

For PCMs applied under cyclic loads, an important performance parameter is fatigue resistance. It depends on a number of factors, particularly the molecular structure and the mechanical properties of the polymer matrix, the size and characteristics of fillers, as well as their bulk distributions and adhesive strength [11,12]. The main part of the fatigue life of particulate PCMs corresponds to the stage of accumulation of scattered damages. Their small sizes make it difficult to control the structural integrity of such PCMs by conventional non-destructive testing means [13]. This fact raises the challenge of early identification of critical discontinuities and quantitative characterization of the fatigue process development.

In polymers, the deformation/fracture mechanisms are determined primarily by the mobility of macromolecules due to the action of external applied loads [14,15]. However, it decreases after loading PCMs with fillers, which increases the elastic modulus, hardness, and yield point but reduces the elongation at break [16]. Depending on the operating conditions of products and applied fatigue modes, both high-strength characteristics and maintenance of a certain level of ductility may be required [17]. In addition, it is necessary to take into account the influence of fillers on the processability of PCMs. Thus, maximizing the contents of reinforcing fillers (or improving the strength properties by increasing the length of short fibers) does not guarantee the achievement of the best deformation response, especially under cyclic loads [18].

When drawing fatigue curves (relationship of stress and the number of cycles to failure) in logarithmic coordinates, fatigue life is satisfactorily approximated by exponential functions, particularly determined by the Coffin–Manson expression for the low-cycle fatigue (LCF) mode (up to 10^4^ cycles) and the Basquin equation for the high-cycle fatigue (HCF) regime (above 10^4^ cycles) [19,20]. The Coffin–Manson expression corresponds to cyclic loads at a constant amplitude, characterized by the involvement of inelastic strain mechanisms, while the Basquin equation is suitable for the elastic range [21]. For PCMs, fatigue strains rarely exceed a few percent, while the failure mode is brittle [12].

In this study, the HCF behavior of PCMs was examined, focusing on elastic strain development. For this purpose, the Basquin equation was applied as the basis for the developed model. It contains two unknown variables (namely, the fatigue strength coefficient and the fatigue strength exponent) that are constants of materials, determined experimentally. The null hypothesis was a suggestion that these variables are functionally dependent on both PCM compositions and their structures. So, the first research task was to determine these dependencies and implement them in the model.

As noted above, some reliable data on functional characteristics of PCMs are needed for improving their fatigue resistance. They include information on durability under given operating conditions, as well as on the nature of changes in fracture mechanics as damage accumulates. The most important parameters are stiffness, which decreases as the fatigue process develops, and inelastic strains (cyclic creep), reflecting deformations of such products during operation [22]. Both of these parameters are critical and can be used as the failure criteria even before reaching the maximum number of loading cycles. Thus, it is necessary to assess the kinetics of changes in these characteristics under cyclic loads to describe the fatigue properties of the PCMs. Typically, their quantitative investigations are carried out by analyzing hysteresis loops [23,24]. Accordingly, the above-mentioned parameters should be considered in the developed model, enabling the prediction of the residual life of products made from PCMs [25,26].

The second task addressed in this study was the justification of an approach for the rational design of PCMs based on polyetherimide (PEI), a thermoplastic HPP, loaded with short carbon fibers (CFs) for improving their fatigue life. In this way, the model was advanced on the basis of some previously published data. As a result, it enables the prediction of the development of such processes when varying the CF content and the level of cyclic loads (the maximum stress in a cycle), both in the studied range of operating conditions (by interpolation) and beyond its limits (by extrapolation). Accordingly, the fatigue behavior can be assessed using a minimum number of experimental results [27].

In a previous paper [28], the authors showed that increasing the length of CFs from 200 μm up to 2000 μm slightly enhanced the durability of similar PEI/CF PCMs. At the same time, raising the length of CFs (up to a few or tens of millimeters) sharply deteriorated their processability, so loading with short CFs (in particular, 200 μm) is rational, allowing agglomeration to be avoided when varying their content within a wide range (10–30 wt.%). On this basis, it was expected that varying the CF content and increasing their length could improve the fatigue properties of the PEI/CF PCMs [29].

From the perspective of developing the fatigue model, and simultaneously considering variations in the maximum stress in a cycle and the CF content, it was extremely important to determine the relationships between these variables and the durability of the PCMs [30]. Taking into account their mutual influence has necessitated the assessment of new interdependencies, previously overlooked within the single-parameter formulations [31]. Since cyclic creep is observed in particulate PCMs under cyclic loads due to the accumulation of fatigue damage [32], it was relevant to consider the ratio of the maximum stress in a cycle to the yield point, as a quantitative parameter characterizing this process [26].

Therefore, the varied input parameters were the CF content and the maximum stress in a cycle, while the output parameter was the fatigue life of the PCMs.

This paper is structured as follows. Section 2 presents the used materials and applied research methods. Section 3.1 provides the results of static tests, while the data obtained in fatigue examinations are described in Section 3.2. Section 3.3 is devoted to assessing the stiffness reduction; cyclic creep data are reported in Section 3.4. The developed model and the results of its implementation are given in Section 3.5. The conclusions are preceded by a discussion of the obtained results (Section 4).

## 2. Materials and Methods

For the fabrication of the PEI/CF PCMs, the R00H powder was used (T&T Industry Group Ltd., Shenzhen, China) with a particle size of 16 µm, loaded with milled short CFs (Tenax^®^-A, Teijin Carbon Europe Gmbh, Wuppertal, Germany) 200 µm long at an aspect ratio of 20. Three types of PCMs containing 10, 20, and 30 wt.% CFs were manufactured by hot pressing (the CF weight fraction denoted as *M*_f_ and equals 0.1, 0.2 and 0.3, respectively).

Static tensile tests were performed according to [33], where 3 specimens tested for each composite. Fatigue tests were carried out in accordance with [34] under the load control (soft) mode using 3 specimens for each composite and stress level. The *σ*_max_ maximum stresses in a cycle were 75, 65, and 55 MPa. A sinusoidal mode was applied for changing the load with an *R* cycle asymmetry coefficient of 0, i.e., the minimum stress was zero in all tests. In the tests, two blocks alternated, namely the main loading at a frequency of 5 Hz and measurement cycles (for recording the parameters of hysteresis loops) at a frequency of 1 Hz. Reducing the loading rate upon the measurement cycles was necessary to obtain sufficient numbers of points to plot the hysteresis loops and calculate their parameters. The main block durations varied from 50 cycles for the PEI/10CF PCM (at *σ*_max_ = 75 MPa) up to 5000 cycles for the PEI/30CF sample (at *σ*_max_ = 55 MPa). They were preset in such a way that 100–200 points were recorded for plotting graphs of the loop parameters during the fatigue test.

The fatigue properties of the PCMs were assessed by calculating the parameters of the mechanical hysteresis loops drawn in the strain–stress coordinates [35]. Stresses were calculated using the data recorded with a load cell of a testing machine. Strains were determined using the (non-contact) digital image correlation (DIC) method [36]. An optical extensometer at gauge length of 7.62 mm and the “VIC 2D 2009” software package (Correlated Solutions, Irmo, SC, USA) were used. The stress and strain data were synchronized using a hardware trigger [37].

A “LEO EVO 50” scanning electron microscope (SEM; Carl Zeiss, Oberkochen, Germany) was applied for examining fracture surfaces after the fatigue tests.

Since the experimental results were not deterministic, they could have a significant random component. In this regard, the task of the obtained data approximation was to show the general trends (the functional components of the general stochastic relationships between the variables). With a limited array of experimental data, such dependencies could be characterized using regression equations in two stages. The first stage was related to selection of the function type, i.e., a class of equations reflecting the correlation between the analyzed variables. In the second stage, the parameters were determined for a specific function, for which it best approximated the experimental results using the least-squares method. Initially, simpler models were tested, but more complex models were applied if convergence was poor. To assess the convergence between the experimentally obtained data and the model parameters, the *R*^2^ coefficient of determination (COD) was applied. Its target value was not less than 0.9.

## 3. Results

### 3.1. Static Tests

**Figure 1:**
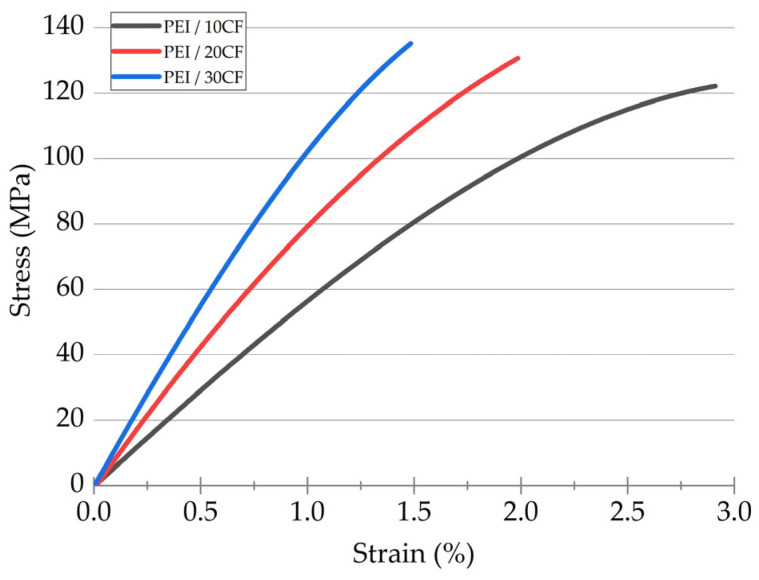
The stain–stress engineering diagrams of the PEI/CF PCMs obtained in the static tensile tests.

**Figure 2:**
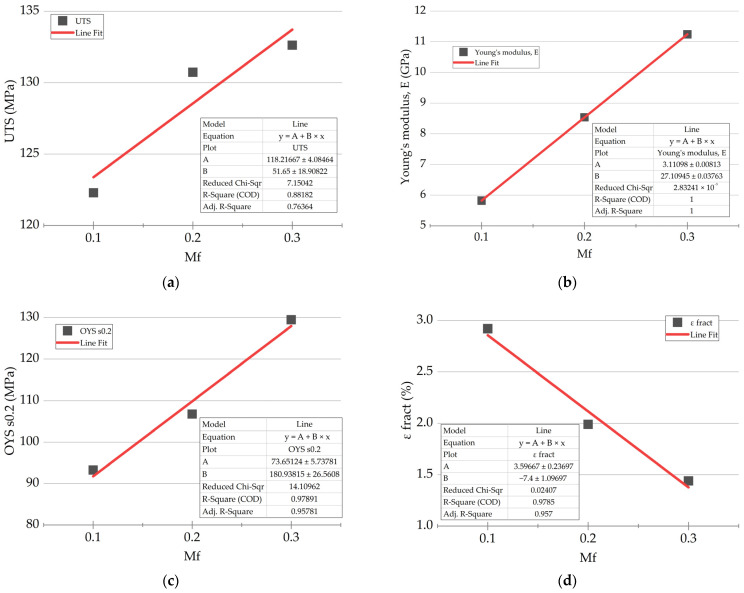
The dependences of the mechanical properties of the studied PCMs vs. the CF content and their linear approximations (red lines): (**a**) ultimate tensile strength (*σ*_UTS_); (**b**) elastic modulus (*E*); (**c**) yield point (*σ*_0.2_); (**d**) elongation at break (*ε*_fract_).

Stain–stress engineering diagrams for the PEI/CF PCMs, obtained in the static tensile tests, are shown in Figure 1, while their mechanical properties are summarized in Table 1. Despite a threefold increase in the CF content (from 10 up to 30 wt.%) in the studied PCMs, their ultimate tensile strength *σ*_UTS_ increased slightly. This phenomenon could be explained by the fact that the strength characteristics were limited primarily by those of the polymer matrix in such cases. According to Figure 2a, the *σ*_UTS_ versus *M*_f_ dependence was not exactly linear (COD was 0.88 after the linear approximation). On the contrary, the elastic modulus *E* versus CF content dependence was linear (Figure 2b). Both the dependences of yield point *σ*_0.2_ and elongation at break *ε*_fract_ were also satisfactorily approximated by the linear function.

The following equation of the *σ*_0.2_ = *f* (*M*_f_) functional dependence was obtained:

(1) $$\sigma_{0.2}=74+181\times M_{f}$$

It followed from Equation (1) that *σ*_0.2_ = 74 MPa in the absence of CFs, which corresponded to the data for neat PEI. The *E* value of 3.11 GPa was correlated as well.

### 3.2. Fatigue Tests

The symbol *σ*_max/0.2_ characterizes the ratio of maximum stress in a cycle (*σ*_max_) over the yield strength (*σ*_0.2_). The results of the fatigue tests and the *σ*_max/0.2_ parameter are given in Table 2.

As mentioned above, the Basquin equation [20] relates the maximum stress in a cycle with the number of cycles to failure:

*σ_max_* = *C* × *N*_f_ ^*n*^,

where *σ*_max_ is the maximum stress in a cycle, *N*_f_ is the number of cycles to failure, *C* is the fatigue strength coefficient, *n* is the fatigue strength exponent, and *C* and *n* are experimentally determined parameters.

For the logarithmic coordinates, the following equation of the linear dependence could be written:

$${\log\left(\sigma_{max}\right)=\log\left(C\right)+n\times log(N}_{f})$$

**Figure 3:**
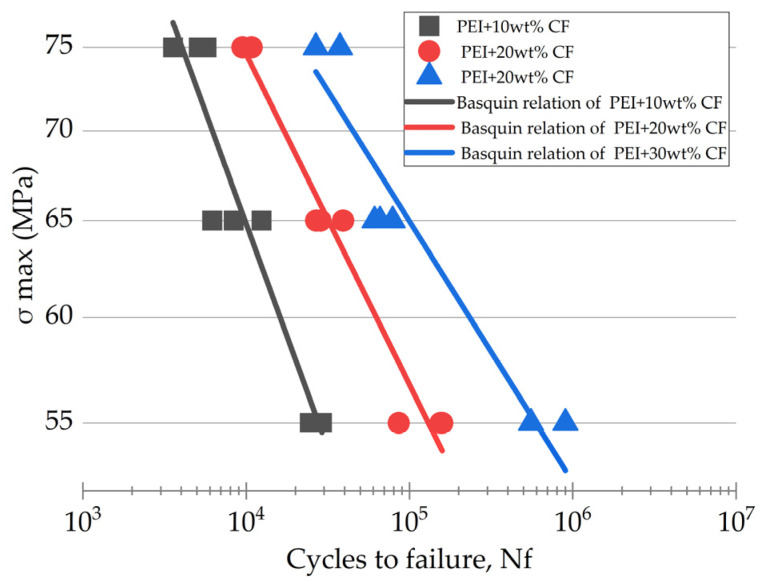
The fatigue curves in the “*σ*_max_ − *N*_f_” coordinates for the PCMs with = CF contents of 10, 20, and 30 wt.%, as well as the approximation lines obtained using the developed model on the basis of the Basquin equation.

Figure 3 shows both the graphical representation of the fatigue test results for the studied PCMs and the data approximated using the Basquin equation. For the PEI/30CF sample, this data processing algorithm did not reflect well the experimentally obtained trend.

**Figure 4:**
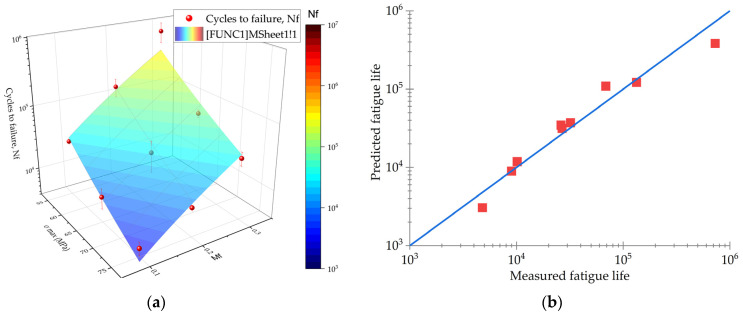
The fatigue test results (red dots) at various combinations of the maximum stresses in a cycle and the CF contents, as well as the trends predicted using the developed model on the basis of the Basquin equation: (**a**) the 3D surface; (**b**) the model convergence, where red squares depict fatigue life for various *M*_f_ and *σ*_max_.

It should be noted that the Basquin equation was applicable for predicting only in the HCF mode (*N*_f_ ≥ 10^4^), since it considered elastic strains. According to Figure 4, its implementation led to an underestimation of the predicted durability relative to the experimentally determined data at both *N*_f_ ≤ 10^4^ and *N*_f_ ≥ 10^5^.

Some models on the basis of the Basquin equation were modified by many authors previously, for example, by Niesłony et al. [38] and Yang et al. [39]. They demonstrated that the fatigue strength coefficient and the fatigue strength exponent were linearly dependent on the yield points, but these methods were applied mainly in studying metals loaded at constant strain amplitudes under severe conditions. In logarithmic coordinates, S–N curves were characterized by linear dynamics, but such dependences might not be observed for PCMs.

**Figure 5:**
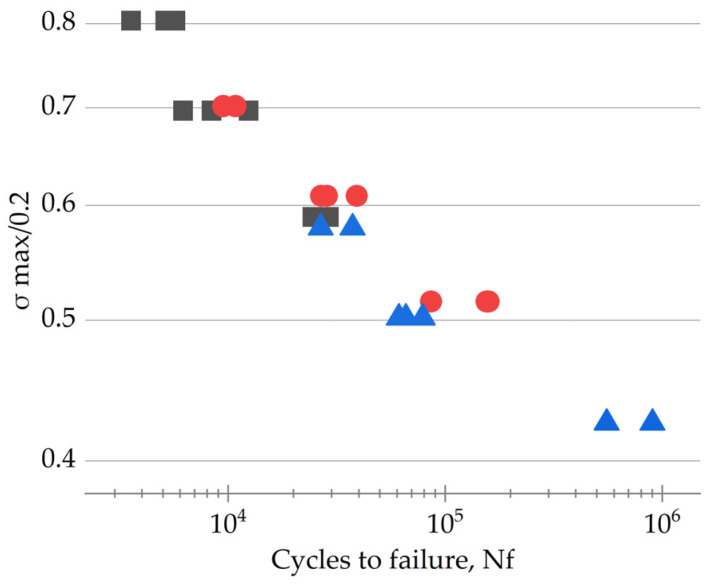
The results of the fatigue tests presented in “*σ*_max/0.2_ − *N*_f_” coordinates for the PCMs with the CF contents of 10 (black points), 20 (red points), and 30 wt.% (blue points).

Based on the hypothesis that the inelastic strain development was the key indicator of the accumulation of fatigue damages in the particulate PCMs, and the yield points determined the proportions of inelastic strains per cycle, it could be concluded that changing the yield points of the PCMs by varying their CF contents was equivalent in nature to varying the maximum stress in a cycle. These assumptions were confirmed by the experimental data presented in Figure 5. In the studied cases, the fatigue test results drawn in the “*σ*_max/0.2_ − *N*_f_” coordinates could be satisfactorily approximated by a single straight line.

The PEI/30CF sample was characterized by the lowest *σ*_max/0.2_ ratio, inhibiting the inelastic strain development and improving durability. In logarithmic coordinates, fatigue life did not obey the linear law predicted using the model by the Basquin equation, and it had the greatest deviations from it at both high and low *σ*_max/0.2_ ratios.

To improve the model convergence, the model was modified in three ways:

1. For considering the influence of inelastic strains, the *σ*_max/0.2_ ratios were implemented instead of the maximum stress in a cycle;
2. The parabolic *σ*_max_ versus *N*_f_ dependence was used in logarithmic instead of linear coordinates;
3. The *N*_f_ versus *σ*_max_ dependence was applied initially.

So, the modified model by the Basquin equation was as follows:

$$\log\left(N_{f}\right)=C+n\times \log\left(\sigma_{max/0.2}\right)+m\times {\left(\log\left(\sigma_{max/0.2}\right)\right)}^{2},$$

or

$$N_{f}=10^{C+n\times \log\left(\sigma_{max/0.2}\right)+m\times {\left(\log\left(\sigma_{max/0.2}\right)\right)}^{2}}.$$

**Figure 6:**
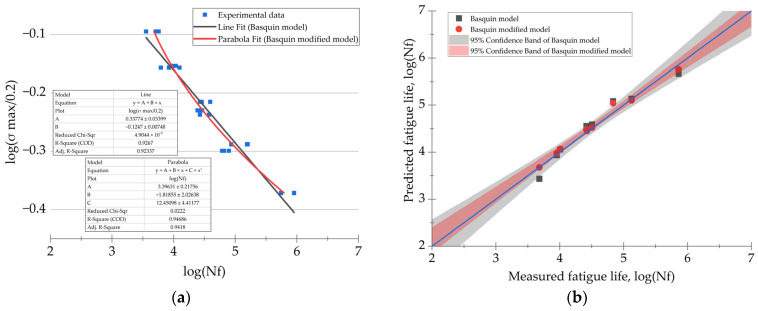
The results of the data approximation using both original and modified models by the Basquin equation: (**a**) the fatigue curves; (**b**) convergence of the models with the experimental data.

Figure 6 shows two lines approximated using both original and modified models. The modified model (red curve in Figure 6a) more accurately reflected the experimental data, while its confidence interval was narrower (Figure 6b), improving the accuracy of the durability prediction. In the LCF mode (less than 10^4^ cycles to failure), the higher convergence indicated that the resulting parabolic dependence could be used for prediction in similar cases.

By expressing *σ*_max/0.2_ through *σ*_max_ and *σ*_0.2_ (Equation (1)) and substituting the *C* = 3.4, *m* = 12, and *n* = −2 values, the final equation could be obtained for assessing the durability of the PCMs depending on the maximum stress in a cycle and the CF content:

(2) $$N_{f}=10^{3.4-2\times \log\left(\frac{\sigma_{max}}{74+181M_{f}}\right)+12\times {\left(\log\left(\frac{\sigma_{max}}{74+181M_{f}}\right)\right)}^{2}}$$

During the fatigue tests, hysteresis loops were drawn and their parameters, such as the dynamic modulus and inelastic strains (due to cyclic creep), were calculated. Then, these data were analyzed and a computer simulation of the hysteresis loop parameters was carried out.

### 3.3. Stiffness Reduction

The dynamic modulus was calculated in each measurement cycle from the hysteresis loop parameters according to the following equation:

*E*_dyn_ = (*σ*_max_ − *σ*_min_)/(*ε*_max_ − *ε*_min_)

**Figure 7:**
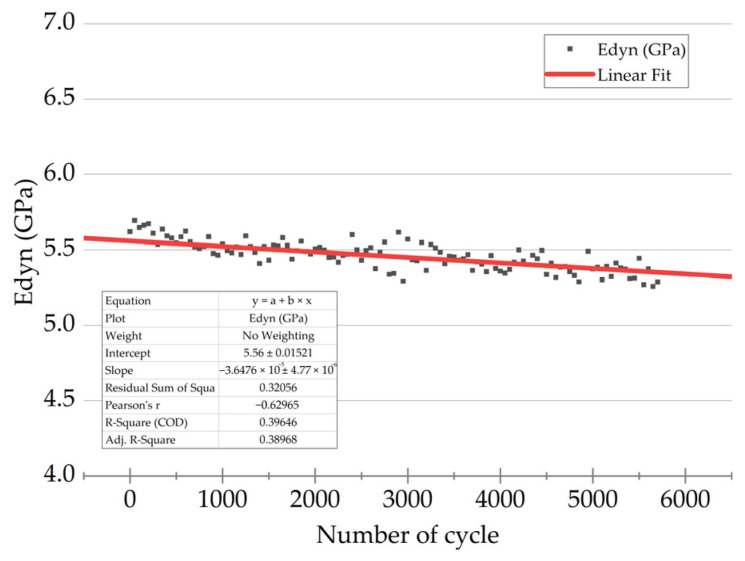
A typical “*E*_dyn_ versus number of cycles” dependence and its linear approximation (a red line).

As a result, “*E*_dyn_ versus number of cycles” dependencies were drawn for the studied PCMs. Figure 7 shows an example for the PEI/CF10 sample at the maximum stress in a cycle of 75 MPa. Most dependences exhibited a linear pattern. Accordingly, the obtained data were linearly approximated to simplify the perception of information and to identify the general trends.

An equation of the linear approximation contained two terms: the initial value (at *N* = 0) and the slope angle, reflecting the *E*_dyn_ changing rate (designated as *V*_dyn_). So, the equation was as follows:

*E*_dyn_ = *E*_dyn first_ + *V*_dyn_ × *N* (3)

At the fracture point (*E*_dyn Nf_), the *E*_dyn_ value was determined by substituting the number of cycles to failure into the resulting equation. The *E*_dyn loss_ parameter was defined as the difference between the initial *E*_dyn first_ and final *E*_dyn_ (at the fracture point) values. The obtained values are summarized in Table 3.

**Figure 8:**
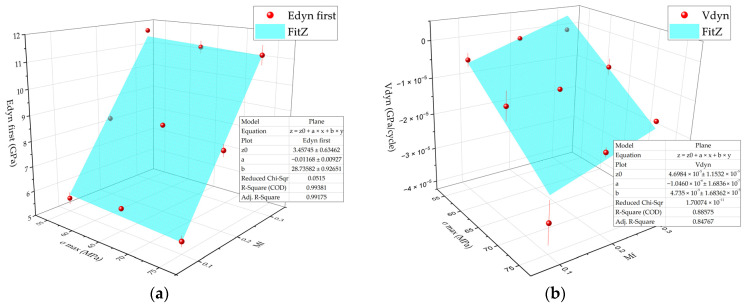
The dependences of the *E*_dyn_ parameters versus the maximum stress in a cycle and the CF content (red dots correspond to the experimental data) and the blue surfaces drawn using the *z = z*0 *+ a × x + by* equation: (**a**) the *E*_dyn first_ values; (**b**) the *V*_dyn_ values.

The dependence of the initial *E*_dyn first_ dynamic modulus on the *σ*_max_ and *M*_f_ values was described well by a plane equation (Figure 8a):

(4) $$E_{dyn\;first}=3.5-0.012\sigma_{max}+28.7M_{f}$$

The minus sign of the *σ*_max_ coefficient reflected the fact that its growth increased the plastic component but decreased the dynamic modulus. At the same time, the influence of the CF contents on the *E*_dyn_ values was more significant than that for *σ*_max_ levels; thus, the *M*_f_ coefficient was much greater. It followed from Equation (4) that the dynamic modulus of the PEI/CF PCM was 3.3 GPa at both zero stress in a cycle and zero CF content, which was close to that for neat PEI.

The dynamics of changes in the dynamic modulus is shown in Figure 8b as a surface. Raising the maximum stress in a cycle decreased its values, while they enhanced with increasing CF content.

The *V*_dyn_ rate of changes in the dynamic modulus from the CF content and the maximum stress in a cycle was described by the following equation:

(5) $$V_{dyn}=4.7\times 10^{-5}-1.05\times 10^{-6}\times \sigma_{max}+4.7\times 10^{-5}\times M_{f}$$

Substituting (4) and (5) into (3), the dependence of the dynamic modulus on the cycle number, the maximum stress in a cycle, and the CF content was written:

(6) $$E_{dyn}=3.5-0.012\sigma_{max}+28.7M_{f}+\left(4.7\times 10^{-5}-1.05\times 10^{-6}\times \sigma_{max}+4.7\times 10^{-5}\times M_{f}\right)\times N$$

It enabled the estimation of the level of decrease in the dynamic modulus when a given number of cycles was reached, depending on the maximum stress in a cycle and the CF content in the PCMs.

After substituting Equation (2) into (6), the following equation was formulated to determine the dynamic modulus at the fracture point (*N* = *N*_f_):

(7) $$E_{dyn}=3.5-0.012\sigma_{max}+28.7M_{f}+\left(4.7\times 10^{-5}-1.05\times 10^{-6}\times \sigma_{max}+4.7\times 10^{-5}\times M_{f}\right)\times 10^{3.4-2*\log\left(\frac{\sigma_{max}}{74+181M_{f}}\right)+12\times {\left(\log\left(\frac{\sigma_{max}}{74+181M_{f}}\right)\right)}^{2}}$$

**Figure 9:**
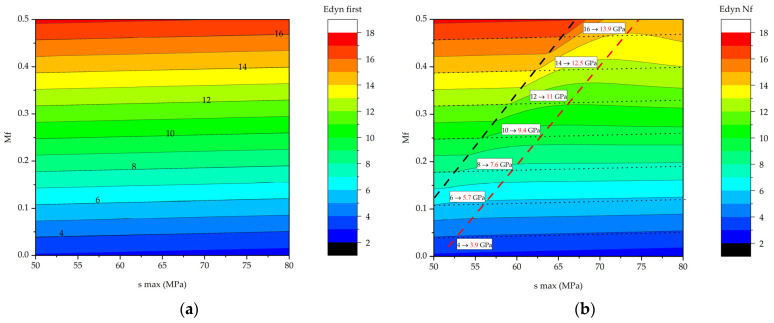
The calculated dependences of the dynamic modulus on the maximum stress in a cycle and the CF content, presented graphically as surfaces: (**a**) at the beginning of the tests; (**b**) at the fracture point. Red numbers depict *E*_dyn_ value at failure.

Figure 9 shows dependences of the dynamic modulus at various combinations of the maximum stress in a cycle and the CF content, calculated both at the beginning of the tests (*N* = 1) according to Equation (6) and at the fracture point (*N* = *N*_f_) using Equation (7). The presented data made it possible to estimate both the initial stiffness level (expressed through the dynamic modulus) and the degree of its reduction at the fracture point. In Figure 9b, an area in the upper left corner is highlighted with a black dashed line in which zero dynamic modulus reduction was observed.

After comparing the data presented in Figure 9a,b, it was concluded that the greatest stiffness reduction was observed for certain maximum stresses in a cycle and CF contents (shown by a red dashed line). These critical *σ*_max_ levels increased with rising *M*_f_ values. The proximity of the red and black dashed lines could be explained as follows: the linear dependencies used to characterize the initial dynamic modulus and the rate of its reduction suggested that the dynamic modulus had to be significantly decreased even at negligible rates of its reduction and very large numbers of cycles to failure. At the same time, the rate of reduction in dynamic modulus tended to zero upon reaching a certain threshold level, when the stiffness reduction was not observed for the marked maximum stresses in a cycle and maximum CF contents. Thus, even a negligible reduction in dynamic modulus in some PCMs with a high calculated durability could result in a substantial stiffness reduction at large number of cycles. So, it is recommended to use compositions from the region characterized by the absence of reduction in the dynamic modulus in designing particulate PCMs.

In general, Equation (6) enabled the determination of current stiffness levels for any numbers of cycles (not exceeding *N*_f_).

### 3.4. Cyclic Creep

The assessment of the inelastic component, developed in the fatigue tests, was carried out by analyzing changes in average strains, calculated using the following equation:

*ε*_mean_ = (*ε*_max_ + *ε*_min_)/2

Accordingly, the inelastic component (*ε*_cc_ cyclic creep) was the difference between the current average strain and that at the first cycle:

*ε*_cc_ = *ε*_mean N_ − *ε*_mean 1_

**Figure 10:**
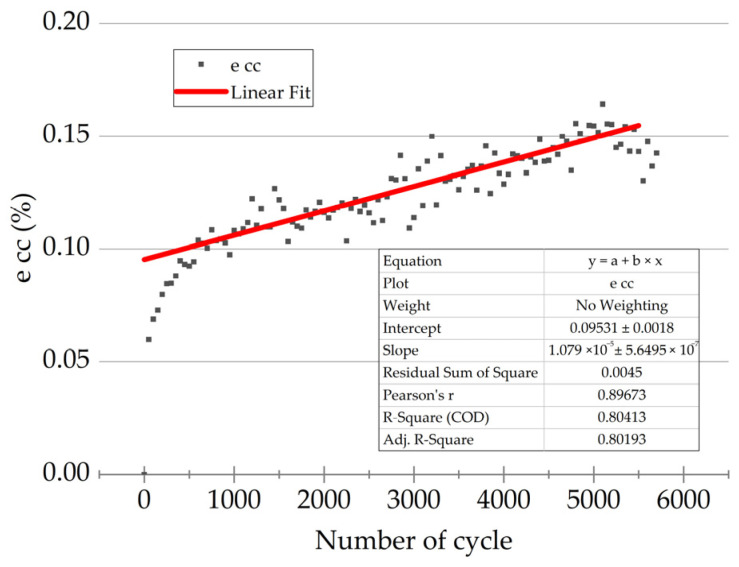
Variations in the *ε*_cc_ values under cyclic loads and the linear approximation (a red line) for the “steady-state” mode of the cyclic creep process.

A typical trend of the *ε*_cc_ values under cyclic loads is presented in Figure 10. During the first 400 cycles, an accelerated increase in inelastic strains was evident, which the authors associated with the breaking-in of the PCMs and the passing of the cyclic creep process into the steady-state mode. The subsequent section of the “*ε*_cc_ − *N*” dependence reflected the second cyclic creep stage, when the strain rate was constant. This linear section was approximated by the *Y = A + B × X* equation and was used to characterize the inelastic strain development in the studied PCMs.

Based on the above, the equation of cyclic creep depending on the number of cycles could be rewritten as

*ε*_cc_ = *ε*_cc first_ + *V*_εcc_ *N*

The *ε*_cc_ values characterized the initial cyclic creep, developed during the first cycles; the *V_ε_*_cc_ creep rate was determined from the slope of the approximation line. At the *N*_f_ fracture point, the *ε*_cc_ value depended on the number of cycles to failure.

Then, linear equations were obtained for all other combinations of the maximum stresses in a cycle and the CF contents. The results are summarized in Table 4.

**Figure 11:**
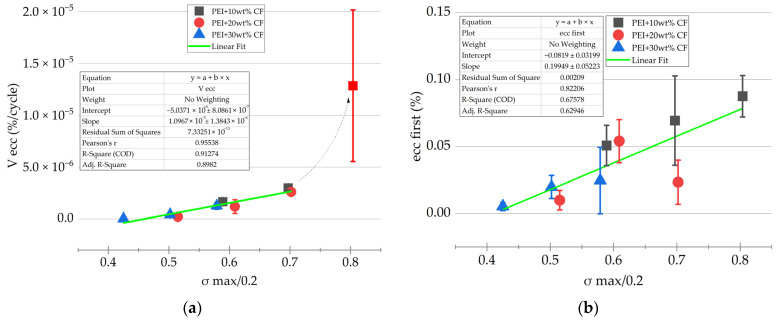
The *ε*_cc first_ and *V*_εcc_ dependences on the *σ*_max/0.2_ values obtained in the fatigue tests, as well as their linear approximation: (**a**) the *V*_εcc_ parameter; (**b**) the *ε*_cc first_ parameter.

The *V*_εcc_ parameter was the most informative, reflecting the rate of inelastic strain development. Assuming that the *V*_εcc_ values depended on the yield points of the PCMs, their dependence on the *σ*_max/0.2_ levels was drawn (Figure 11a). Satisfactory convergence was obtained by the linear approximation in the *σ*_max/0.2_ range of 0.4–0.8. The intersection with the *X* axis was at the *σ*_max/0.2_ level of 0.46, i.e., at the maximum stresses in a cycle below 0.46 of the yield points; the rate of the inelastic strain development was zero. This observation corresponded to the maximum stresses in a cycle of 43, 49, and 60 MPa for PEI/10CF, PEI/20CF, and PEI/30CF PCMs, respectively.

A deviation from linear dependence was also observed at *σ*_max/0.2_ = 0.8, which was most likely caused by reaching the yield point. These conditions were implemented for the PEI/10CF sample at *σ*_max/0.2_ = 75 MPa, when the number of cycles to failure of ~4800 corresponded to the LCF mode with a significant proportion of inelastic strains. Therefore, the *σ*_max/0.2_ parameter could be applied to differentiate the fatigue tests, when ensuring that the HCF mode could be justified at *σ*_max/0.2_ ≤ 0.7.

Similarly, both *ε*_cc first_ and *V*_εcc_ dependences on the *σ*_max/0.2_ values were plotted (Figure 11b) using the following equations:

*ε*_cc first_ = −0.08 + 0.2 × *σ*_max/0.2_

*V*_εcc_ = −5 × 10^−6^ + 10^−5^ × *σ*_max/0.2_

Expressing *σ*_max/0.2_ in terms of *σ*_max_ and *M*_f_:

$$\varepsilon_{cc\;first}=-0.08+0.2\times \left(\frac{\sigma_{max}}{74+181\times M_{f}}\right)$$

$$V_{\varepsilon cc}=-5\times 10^{-6}+10^{-5}\times \left(\frac{\sigma_{max}}{74+181\times M_{f}}\right)$$

The final equation for cyclic creep at a given *N* number of cycles was as follows:

(8) $$\varepsilon_{ccN}=-0.08+0.2\times \left(\frac{\sigma_{max}}{74+181\times M_{f}}\right)+\left(-5\times 10^{-6}+10^{-5}\times \left(\frac{\sigma_{max}}{74+181\times M_{f}}\right)\right)\times N$$

By substituting the number of cycles to failure *N*_f_ (from Equation (2)) in (8), instead of *N*, it was possible to determine inelastic strains (cyclic creep) at the fracture point.

**Figure 12:**
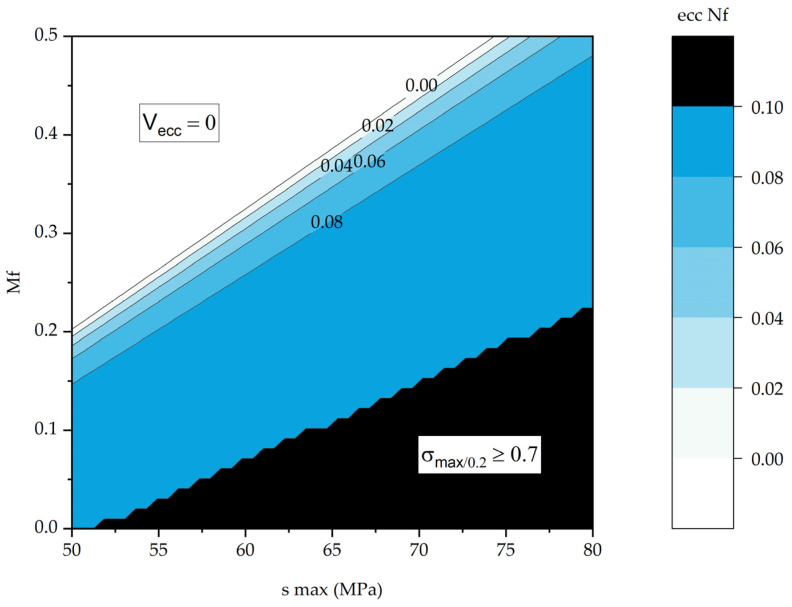
The dependences of the *ε*_cc_ parameters (at *N = N*_f_) on the CF content and the maximum stress in a cycle.

In addition, the previously defined limitation *σ*_max/0.2_ ≤ 0.7 was applied, reflecting the limit below which increased inelastic strains were observed in the LCF mode. The obtained results are graphically presented in Figure 12. Inelastic strains arose due to cyclic creep, increasing with the enhancement in maximum stress in a cycle but decreasing with the increase in the CF content in the PCMs. Three characteristic ranges were identified: the “white” region reflected combinations of the CF contents and the maximum stresses in a cycle, at which the rate of inelastic strain development was zero; the “colored” region in the center marked an area with linear and predictable inelastic strain development; the “black” region characterized the LCF mode, in which inelastic strains developed unstably, so the PCMs were not recommended for applications under such conditions.

### 3.5. Fatigue Simulation

Table 5 presents the fatigue properties of the PEI/CF PCMs, calculated using the developed model at CF contents of 5–50 wt.% and maximum stresses in a cycle of 50–80 MPa. The reported data were formatted in such a way that the durability (*N*_f_) of the PCMs, the stiffness at the beginning of the fatigue tests (*E*_dyn_ at *N* = 1), and its reduction and inelastic strains at the fracture point (*E*_dyn loss_ and *ε*_cc_ at *N* = *N*_f_) could be traced for each CF content and maximum stress in a cycle. The model considered the restrictions where the dynamic modulus could not increase during the fatigue test and inelastic strains could not be negative. Some cases are highlighted in red, in which *σ*_max/0.2_ exceeded 0.7 and increased the inelastic strains characterized by their unstable development, and the LCF mode could be expected. PCMs with the greatest durability at the given maximum stresses in a cycle are marked in green.

An analysis of the results presented in Table 5 enabled the following conclusions to be drawn:

1. In general, PCMs with the highest CF content (50 wt.% in the studied range) were characterized by the greatest durability.
2. Some combinations of maximum stresses in a cycle and the CF contents were identified, which contributed to the unstable development of inelastic strains (according to the data predicted using the developed model) and had to be avoided.
3. Several PCMs were characterized by zero inelastic strain development and zero dynamic modulus reduction at the maximum cycle stresses in a cycle from 50 up to 80 MPa.
4. The CF content significantly affected the fatigue properties of the PCMs and could be effectively used as one of the key criteria in their design.

It was important to note that the stated conclusions did not take into account some possible challenges associated with ensuring uniform distributions of CFs (avoiding their agglomeration) when their contents in the polymer were very high (for example, 55 wt.%, i.e., when the polymer actually was a binder but not a matrix).

**Figure 13:**
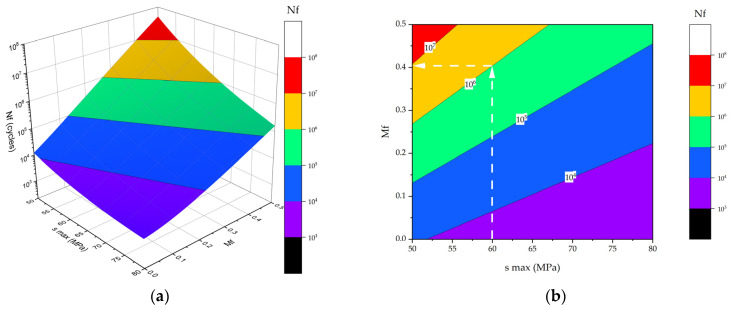
The calculated dependences of durability of the PCMs on the maximum stress in a cycle and the CF content: (**a**) 3D; (**b**) 2D. White broken lines show the principle of selecting the required PCM composition.

Figure 13 shows the calculated dependences of durability of the PCMs on the maximum stress in a cycle and the CF content. They enabled the determination of PCM compositions to ensure their required durability under given cyclic loads. As an instance, Figure 13b justifies the principle of selecting the required PCM composition at the maximum stress in a cycle of 60 MPa: a vertical line was drawn connecting the stress amplitude before the intersection with the isoline of the required durability; then, a horizontal line was plotted until it intersected with the *M*_f_ axis, determining the required CF content. In this example, it was ~40 wt.% CF for achieving a durability of 10^6^ cycles.

An advantage of the developed model was the possibility to predict both fatigue life and deformation behavior of the PEI/CF PCMs within and outside the experimentally studied range of their compositions and the test conditions. This fact enabled the evaluation of an improvement degree for the fatigue properties by varying the CF contents. If the achievable value of the predicted fatigue properties was insufficient, it was advisable to use other modification methods (for example, by changing the polymer matrix and/or filler(s), enhancing the interfacial adhesion level, etc. [22]).

## 4. Discussion

**Figure 14:**
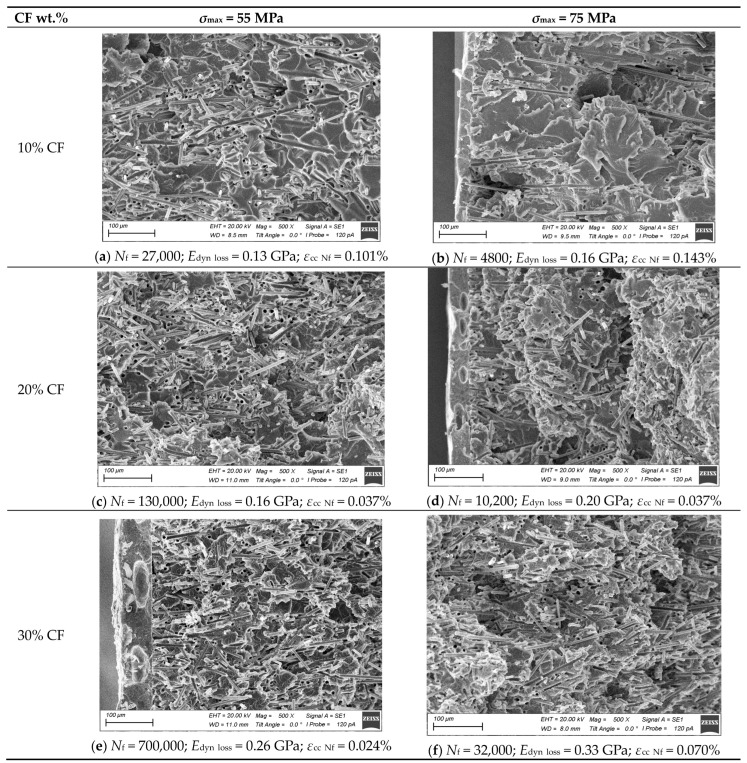
The SEM micrographs of the fracture surfaces on the studied PCMs with the CF contents of 10, 20, and 30 wt.% after the fatigue tests at different maximum stresses in a cycle; a magnification of ×100: (**a**,**c**,**e**) *σ*_max_ = 55 MPa; (**b**,**d**,**f**) *σ*_max_ = 75 MPa.

The obtained results should be discussed by analyzing the structural aspects of the studied PCMs. Figure 14 shows SEM micrographs of their fracture surfaces after the fatigue tests at the maximum cycle stresses in a cycle of 55 and 75 MPa. These levels of the cyclic loads exerted a pronounced effect on the fracture mechanisms and the examined surfaces, since they were above and below the yield point of the polymer matrix. At *σ*_max_ = 75 MPa, great inelastic strains were present in the matrix regardless of the CF contents, which was expressed by a wavy and rough relief on the fracture surfaces.

According to Figure 14, the fracture surfaces became less uniform with increasing CF content in general, which was associated with branching of the main crack trajectories. Greater amounts of CFs prevented their propagation, raising the amount of expended energy and improving the fatigue resistance of the PCMs.

However, as noted above, the stage of propagation of a main crack constituted a smaller part of the fatigue process, while the main contribution to durability was determined by the period of accumulation of scattered damages. The latter had to predominantly arise at the polymer–CF interface due to the multiple differences in their elastic moduli. In this regard, the studied PCMs were defined as particulate (dispersedly filled), when the CFs locally modified the polymer matrix, along which the main cracks propagated, determining the pattern of the fracture processes. On the other hand, it was dispersion strengthening, regardless of taking into account the adhesion factor, that provided such a significant difference in fatigue life when changing both the CF content and the maximum stress in a cycle.

Also, it was found that increasing the maximum stress in a cycle from 55 up to 75 MPa changed the fracture surface patterns. At low CF contents (Figure 14a,b) and the high *σ*_max_ level of 75 MPa, the basis of the fracture surfaces included mostly the polymer matrix (giving the impression that the PCMs were locally “depleted” of CFs), while greater amounts of CFs were found at *σ*_max_ = 55 MPa.

Increasing the CF content up to 20 wt.% changed the above-described trend. At *σ*_max_ = 75 MPa, the uneven propagation of the main crack contributed to the formation of a terrace relief (Figure 14d). The reason for such a phenomenon could be the presence of a transition period between the LCF and HCF modes. At *σ*_max_ = 55 MPa, raising the CF content slowed down the cyclic creep process, which reflected the degree of mobility of macromolecules and the ability of the polymer matrix to undergo structural rearrangement. For example, increasing the CF content from 10 up to 30 wt.% reduced the number of “weak points”, i.e., regions in the PCMs depleted of CFs, in which intense inelastic strains, fatigue damage, and the main crack were observed. Thus, fatigue life was prolonged in this case. For PCMs with different CF contents, inelastic strains differed at their fracture points, but their failure occurred after reaching similar cyclic creep levels at various maximum stresses in a cycle but the same CF content.

Based on the above, it could be concluded that cyclic creep was a parameter sensitive to the number of “weak points” of the polymer matrix in which inelastic strain development was possible. Reaching a certain level was critical and could indicate the initiation of a main crack. Thus, the critical strain levels were 0.12% for the PEI/10CF PCM, 0.049% for the PEI/20CF sample, and 0.046% for the PEI/30CF sample under the cyclic creep conditions.

In the fatigue tests, the matrix–CF interfaces were gradually deteriorated, reducing the load transfer to CFs, which was reflected in the reduction in dynamic modulus. At the same time, raising the CF content increased both the volumetric number of such interfaces and the range of reduction in dynamic modulus without damage, thereby improving the durability of the PCMs. This fact was confirmed by the reduction in dynamic modulus of 0.13, 0.16, and 0.26 GPa (at *σ*_max_ = 55 MPa) and 0.16, 0.20, and 0.33 GPa (at *σ*_max_ = 75 MPa) for the PCMs with 10, 20, and 30 wt.% CFs, respectively. At *σ*_max_ = 75 MPa, the greater decrease in dynamic modulus could be explained by the fact that inelastic strains were developed and the polymer matrix was fractured in addition to the deterioration in the matrix–CF interfaces under such loading conditions, resulting in the accumulation of another type of discontinuity and reducing the stiffness of the PCMs. Consequently, the dynamic modulus was a parameter sensitive to the deterioration in the matrix–CF interfaces.

It was possible to identify two different mechanisms in the development of such processes. The first mechanism was fracture at the “weak points” of the polymer matrix, characteristic of the PEI/10CF PCM, which determined its low durability. The second mechanism caused failure via the accumulation of damages at the matrix–CF interfaces that was observed in the PEI/30CF sample, improving its durability. The first case was characterized by the least reduction in dynamic modulus and the greatest inelastic strain development. In the second case, on the contrary, the reduction in dynamic modulus was greatest, but inelastic strains were lowest. Accordingly, the “weak points” of the polymer matrix were a critical and limiting fatigue parameter, and without removal, it was impossible to achieve high durability of the PCMs. The adhesion factor (not considered in this study) could influence the second mechanism but could not change the first. As a consequence, loading the PCMs with shorter CFs was preferred due to their more dispersed distribution and reduction in the number of “weak points”.

Therefore, the simultaneous analysis of both strength and deformation parameters enabled the reliable interpretation of the nature of changes in fatigue resistance of the studied PCMs.

From the standpoint of practical applications of the obtained results, the authors considered it necessary to note that the studied PEI/CF PCMs were dispersedly filled with CFs 200 μm long. Such CFs were too short for substantial improvement in the strength properties of the PCMs, but their length was enough to prolong fatigue life by an order of magnitude via raising the CF content. At the same time, these CFs were of the high-tech type, ensuring the possibility of using conventional production routes for their processing, isotropic properties, minimal agglomeration, and some other advantages. In the case of increasing the length of CFs, the patterns of influence of both their contents and test conditions on the durability of the PCMs would be changed, while the aspect of uniformity in the distribution of CFs throughout the volume of samples/parts to ensure load-bearing capacity could be critical.

## 5. Conclusions

In this study, the fatigue behavior of the PEI-based PCMs dispersedly filled with CFs 200 μm long was investigated under the cyclic loads. The varied parameters were the CF content and the maximum stress in a cycle. As a result, the correlation between the yield point of the polymer matrix and fatigue life of the PCMs was shown. The dependences of the elastic modulus, yield point, dynamic modulus, and cyclic creep parameters on the maximum stress in a cycle and the CF content were determined and drawn using the linear approximation.

Inelastic strains, caused by the cyclic creep development, increased with the rise in the maximum stress in a cycle but decreased with the enhancement in the CF content in the PCMs. Three characteristic ranges of this parameter were identified. The first range reflected the combination of the PCM compositions and the maximum stresses in a cycle, at which the rate of the inelastic strain development was zero, typical for loads not exceeding 0.05 of the yield points of the PCMs. The second range characterized the linear predictable development of inelastic strains. The third range was typical for the loads exceeding 0.7 of the yield points of the PCMs and was associated with the LCF mode, in which cyclic creep developed unstably, so the PCMs were not recommended for applications under such conditions. The greatest inelastic strain of 0.143% was observed at the minimum CF content of 10% and the maximum stress in a cycle of 75 MPa, while the most negligible was 0.024% at CF content = 30% and *σ*_max_ = 55 MPa. The greatest decrease in dynamic modulus of 0.33 GPa was determined for the strongest PEI/30CF PCM at *σ*_max_ = 75 MPa.

Based on the obtained experimental results, the fatigue model (developed by the Basquin equation) was modified, which considered both the maximum stress in a cycle and the CF content in the PCMs. It was shown that the model could predict the durability of the PEI/CF PCMs at a COD level of 0.95 (relative to the experimental data). It was found that the parabolic dependence of the number of cycles to failure on the maximum stress in a cycle was more suitable than the linear relationship for the studied PCMs in both LCF and HCF modes. The implemented model made it possible to predict both fatigue life and deformation behavior of the PCMs within and outside the experimentally studied ranges of their compositions and the test conditions. This fact enabled the improvement in the fatigue properties by varying the CF content in the PCMs. If the achieved value of the predicted fatigue properties was insufficient, it was advisable to apply other modification methods (by changing the polymer matrix and/or the filler(s), by increasing the interfacial adhesion level, etc.). The application of the developed model showed the possibility of improving the durability of the PCMs up to 4.41·10^7^ at a CF content of 50 wt.% (with minimal inelastic strains and stiffness reduction).

Finally, the “algorithm” for designing PCMs dispersedly filled with short CFs according to the fatigue life criterion was proposed: the maximum stress in a cycle and durability were assigned as target indicators. So, the surface was drawn in terms of the “CF content–load–durability” interdependence, where the cross-section at a given level of durability determined the required CF content.

The approaches to determine relationships between the composition, structure, and properties of PCMs described in this study can be applied to further expand the model based on the Basquin equation and to improve its versatility using some examples of other thermoplastics and reinforcing inclusions. In this study, a number of important structural parameters were not considered, including interfacial adhesion, whose influence on durability should be understood. The development of such models will make it possible to design PCMs with required properties and to evaluate their possible operating conditions.

## Figures and Tables

**Table 1:** The mechanical properties of the PEI/CF PCMs obtained in the static tensile tests.

Sample	*M*_f_	*σ*_UTS_ (MPa)	*E* (GPa)	*σ*_0.2_ (MPa)	*ε*_fract_, %
PEI/10CF	0.1	122.3 ± 1.5	5.80 ± 0.14	93.3 ± 0.7	2.9 ± 0.1
PEI/20CF	0.2	130.7 ± 2.1	8.5 ± 0.2	106.8 ± 0.8	2.00 ± 0.14
PEI/30CF	0.3	132.6 ± 6.9	11.2 ± 0.9	129.5 ± 2.7	1.4 ± 0.3

± is the standard deviation of the measurements.

**Table 2:** The fatigue properties of the PEI/CF PCMs (the experimental data).

Sample	*σ*_max_ (MPa)	*σ*_max/0.2_	*N*_f_	SD
PEI/10CF	75	0.804	4800	1100
PEI/10CF	65	0.697	9000	3000
PEI/10CF	55	0.589	27,000	2000
PEI/20CF	75	0.702	10,200	900
PEI/20CF	65	0.609	26,000	13,000
PEI/20CF	55	0.515	130,000	40,000
PEI/30CF	75	0.579	32,000	8000
PEI/30CF	65	0.502	69,000	9000
PEI/30CF	55	0.425	700,000	200,000

SD is the standard deviation of the measurements.

**Table 3:** The *E*_dyn_ parameters obtained in the fatigue tests (the experimental data).

No.	*σ*_max_ (MPa)	*M*_f_	*σ*_max/0.2_	*E*_dyn first_ (GPa)	SD	*V*_dyn_ (GPa/cycle)	SD	*E*_dyn Nf_ (GPa)	SD	*E*_dyn loss_ (GPa)	SD
1	75	0.1	0.804	5.49	0.22	−3.40 × 10^−5^	6.02 × 10^−6^	5.33	0.20	−0.16	0.01
2	65	0.1	0.697	5.88	0.10	−1.16 × 10^−6^	3.93 × 10^−6^	5.77	0.19	−0.12	0.09
3	55	0.1	0.589	5.54	0.24	−4.99 × 10^−6^	1.84 × 10^−6^	5.40	0.30	−0.13	0.06
4	75	0.2	0.702	8.12	0.24	−2.00 × 10^−5^	1.29 × 10^−6^	7.92	0.24	−0.20	0.01
5	65	0.2	0.609	8.53	0.11	−9.96 × 10^−6^	4.72 × 10^−7^	8.22	0.12	−0.32	0.08
6	55	0.2	0.515	8.30	0.14	−1.16 × 10^−6^	8.06 × 10^−7^	8.14	0.12	−0.16	0.14
7	75	0.3	0.579	11.22	0.37	−1.53 × 10^−5^	2.77 × 10^−7^	10.72	0.25	−0.33	0.30
8	65	0.3	0.502	11.25	0.25	−6.44 × 10^−6^	2.30 × 10^−7^	10.81	0.22	−0.44	0.15
9	55	0.3	0.425	11.68	0.03	−3.99 × 10^−7^	2.45 × 10^−7^	11.42	0.05	−0.26	0.08

SD is the standard deviation of the measurements.

**Table 4:** The *ε*_cc_ values obtained in the fatigue tests (the experimental data).

No.	*σ*_max_ (MPa)	*M*_f_	*σ*_max/0.2_	*ε*_cc first_ (%)	SD	*V*_εcc_ (%/Cycle)	SD	*ε*_cc Nf_ (%)	SD
1	75	0.1	0.804	0.090	0.014	1.23 × 10^−5^	8.63 × 10^−6^	0.143	0.041
2	65	0.1	0.697	0.077	0.030	3.90 × 10^−6^	2.31 × 10^−6^	0.112	0.024
3	55	0.1	0.589	0.050	0.008	1.89 × 10^−6^	2.39 × 10^−7^	0.101	0.014
4	75	0.2	0.702	0.003	0.003	3.33 × 10^−6^	9.19 × 10^−7^	0.037	0.009
5	65	0.2	0.609	0.027	0.023	2.10 × 10^−6^	1.03 × 10^−6^	0.073	0.019
6	55	0.2	0.515	0.008	0.026	2.17 × 10^−7^	5.93 × 10^−8^	0.037	0.026
7	75	0.3	0.579	0.026	0.010	1.45 × 10^−6^	6.43 × 10^−7^	0.070	0.000
8	65	0.3	0.502	0.012	0.015	4.49 × 10^−7^	3.11 × 10^−7^	0.043	0.010
9	55	0.3	0.425	0.011	0.013	1.66 × 10^−8^	7.22 × 10^−9^	0.024	0.023

SD is the standard deviation of the measurements.

**Table 5:** The fatigue properties of the PEI/CF PCMs (the calculated data).

*σ*_max_ (MPa)	Fatigue Properties	PEI/5CF	PEI/10CF	PEI/20CF	PEI/30CF	PEI/40CF	PEI/50CF
80	*N*_f_ (cycles)	2.73 × 10^3^	3.69 × 10^3^	8.13 × 10^3^	2.07 × 10^4^	5.64 × 10^4^	1.59 × 10^5^
	*E*_dyn_, *N* = 1 (GPa)	3.79	5.25	8.16	11.07	13.98	16.89
	*E*_dyn loss_, *N* = *N*_f_ (GPa)	0.11	0.13	0.23	0.46	0.89	1.48
	*ε*_cc_, *N* = *N*_f_ (%)	0.13	0.11	0.08	0.07	0.06	0.00
70	*N*_f_ (cycles)	4.12 × 10^3^	6.44 × 10^3^	1.82 × 10^4^	5.72 × 10^4^	1.88 × 10^5^	6.23 × 10^5^
	*E*_dyn_, *N* = 1 (GPa)	3.91	5.37	8.28	11.19	14.10	17.01
	*E*_dyn loss_, *N* = *N*_f_ (GPa)	0.12	0.16	0.33	0.67	0.98	0
	*ε*_cc_, *N* = *N*_f_ (%)	0.10	0.09	0.07	0.06	0	0
60	*N*_f_ (cycles)	8.35 × 10^3^	1.54 × 10^4^	5.82 × 10^4^	2.33 × 10^5^	9.45 × 10^5^	3.79 × 10^6^
	*E*_dyn_, *N* = 1 (GPa)	4.03	5.49	8.40	11.31	14.22	17.13
	*E*_dyn loss_, *N* = *N*_f_ (GPa)	0.14	0.22	0.44	0.26	0	0
	*ε*_cc_, *N* = *N*_f_ (%)	0.08	0.07	0.05	0	0	0
50	*N*_f_ (cycles)	2.65 × 10^4^	5.96 × 10^4^	3.16 × 10^5^	1.69 × 10^6^	8.82 × 10^6^	4.41 × 10^7^
	*E*_dyn_, *N* = 1 (GPa)	4.15	5.61	8.52	11.43	14.34	17.25
	*E*_dyn loss_, *N* = *N*_f_ (GPa)	0.18	0.21	0	0	0	0
	*ε*_cc_, *N* = *N*_f_ (%)	0.07	0.05	0	0	0	0

## Data Availability

Data are contained within this article.
